# Penetration of polar organic compounds through the blood-brain barrier

**DOI:** 10.1186/1471-2210-11-S2-A51

**Published:** 2011-09-05

**Authors:** Huba Kalász, Kamil Musílek, Kornélia Tekes

**Affiliations:** 1 Department of Pharmacology and Pharmacotherapy, Semmelweis University, 1089 Budapest, Hungary; 2 Department of Toxicology, University of Defence, 500 01 Hradec Králové, Czech Republic; 3 Department of Pharmacodynamics, Semmelweis University, 1089 Budapest, Hungary

## Background

The effect of polar organic xenobiotics in the central nervous system depends on blood-brain barrier (BBB) penetration of these compounds. Newly synthesized pyridinium aldoximes (K-compounds) are promising antidotes for organophosphate intoxications. However, being highly polar, their BBB penetration is questionable. Using an *in vivo* model we aimed to characterize the BBB penetration of K-compounds.

## Methods

Male Wistar rats were injected intramuscularly with various doses of pyridinium aldoximes, blood, cerebrospinal fluid (CSF), and brain samples were collected after 5, 15, 30, 60 and 180 min. A recently developed and optimized RP-HPLC method was used for analysis. Samples of brain homogenate, blood serum and CSF were subjected to clean-up using precipitation by perchloric acid (pH < 1) and centrifugation at 14,000 rpm at 4°C for 20 min. Before load onto Zorbax Rx-C18 stationary phase, the pH of the supernatants was adjusted to 2. As mobile phase a mixture of acetonitrile and aqueous buffer pH 4.5, also containing ion-pairing agent, was used.

## Results

Dose- and time-dependent BBB penetration of pyridinium aldoximes was experimentally found.

## Conclusions

Dose- and time-dependent brain and CSF levels of these highly polar K-compounds following intramuscular administration suggest contribution of active transport or specific transporters in their BBB penetration. The BBB transport may also depend on the size and charge of the solutes.

